# Optimization of Upper Extremity Rehabilitation by Combining Telerehabilitation With an Exergame in People With Chronic Stroke: Protocol for a Mixed Methods Study

**DOI:** 10.2196/14629

**Published:** 2020-05-21

**Authors:** Dorra Rakia Allegue, Dahlia Kairy, Johanne Higgins, Philippe Archambault, Francois Michaud, William Miller, Shane Norman Sweet, Michel Tousignant

**Affiliations:** 1 School of Rehabilitation Université de Montréal Montreal, QC Canada; 2 The Centre for Interdisciplinary Research in Rehabilitation of Greater Montreal Institut universitaire sur la réadaptation en déficience physique de Montréal Montreal, QC Canada; 3 Mission Universitaire de Tunisie Montreal, QC Canada; 4 McGill University Montreal, QC Canada; 5 Université de Sherbrooke Sherbrooke, QC Canada; 6 University of British Columbia Vancouver, BC Canada

**Keywords:** stroke, rehabilitation, virtual reality, telerehabilitation, upper extremity, motivation

## Abstract

**Background:**

Exergames have the potential to provide an accessible, remote approach for poststroke upper extremity (UE) rehabilitation. However, the use of exergames without any follow-up by a health professional could lead to compensatory movements during the exercises, inadequate choice of difficulty level, exercises not being completed, and lack of motivation to pursue exercise programs, thereby decreasing their benefits. Combining telerehabilitation with exergames could allow continuous adjustment of the exercises and monitoring of the participant’s completion and adherence. At present, there is limited evidence regarding the feasibility or efficacy of combining telerehabilitation and exergames for stroke rehabilitation.

**Objective:**

This study aims to (1) determine the preliminary efficacy of using telerehabilitation combined with exergames on UE motor recovery, function, quality of life, and motivation in participants with chronic stroke, compared with conventional therapy (the graded repetitive arm supplementary program; GRASP); (2) examine the feasibility of using the technology with participants diagnosed with stroke at home; and (3) identify the obstacles and facilitators for its use by participants diagnosed with stroke and stroke therapists and understand the shared decision-making process.

**Methods:**

A mixed methods study protocol is proposed, including a randomized, blinded feasibility trial with an embedded multiple case study. The intervention consists of the provision of a remote rehabilitation program, during which participants will use the Jintronix exergame for UE training and the Reacts Application to conduct videoconferenced sessions with the therapists (physical or occupational therapists). We plan to recruit 52 participants diagnosed with stroke, randomly assigned to a control group (n=26; 2-month on-paper home exercise program: the GRASP with no supervision) and an experimental group (n=26; 2-month home program using the technology). The primary outcome is the Fugl-Meyer UE Assessment, a performance-based measure of UE impairment. The secondary outcomes are self-reported questionnaires and include the Motor Activity Log-28 (quality and frequency of use of the UE), Stroke Impact Scale-16 (the quality of life), and Treatment Self-Regulation Questionnaire (motivation). Feasibility data include process, resources, management, and scientific outcomes. Qualitative data will be collected by interviews with both participants and therapists.

**Results:**

At present, data collection was ongoing with one participant who had completed the exergame- telerehabilitation based intervention. We expect to collect preliminary efficacy data of this technology on the functional and motor recovery of the UE, following a stroke; collect feasibility data with users at home (adherence, safety, and technical difficulties); and identify the obstacles and facilitators for the technology use and understand the shared decision-making process.

**Conclusions:**

This paper describes the protocol underlying the study of a telerehabilitation-exergame technology to contribute to understanding its feasibility and preliminary efficacy for UE stroke rehabilitation.

**Trial Registration:**

ClinicalTrials.gov NCT03759106; http://clinicaltrials.gov/show/NCT03759106.

**International Registered Report Identifier (IRRID):**

DERR1-10.2196/14629

## Introduction

### Background

In up to 85% of stroke survivors, sequelae persist in the upper extremity (UE) [1], resulting in a long-term impact on daily living activities [2,3]. To stimulate neuroplastic changes that promote motor recovery, stroke survivors should follow an intensive, task-specific, stimulating, and, above all, repetitive exercise program [4]. However, the programs offered by conventional therapies, in particular, during chronic stage, are not sufficient for people to achieve the level of repetition and intensity required for recovery [4]. In Canada, patients diagnosed with stroke receive the Graded Repetitive Arm Supplementary Program (GRASP) [5] as a home exercise program after discharge from traditional rehabilitation services [3]. The GRASP is recommended by the Canadian Best Practice Recommendations for Stroke Care [3] to provide training and increase the use of the impaired arm in daily life activities [5]. However, patients must be motivated enough to do the exercises on their own and to perform enough repetitions to achieve improvement. A minimum of 15 hours is suggested for an intervention to result in a moderate improvement in daily living activities following a stroke [6]. Numerous studies offering an intensive exercise program, in chronic stroke, reported significant improvements in UE impairment (*P*=.05) [7,8] and even maintenance of these changes over a period of 6 months after treatment [8].

### Increasing Training Intensity in Stroke Rehabilitation

An interesting alternative that has been proposed to exercise intensity and exploit neuroplastic properties involves the use of virtual reality. For every 30 repetitions performed in a standard rehabilitation session, the same person can perform 600 to 800 repetitions within a 1-hour session in a virtual environment [4]. This is an important difference between the two approaches that highlights the potential of virtual reality. Thanks to its design, a virtual environment can simulate the real world while maintaining total control over the parameters of the tasks to be executed within it to foster motor learning [4]. In the context of rehabilitation, virtual reality has been integrated in the form of *exergames* because the gaming goal is to enhance activity through various types of exercises. Among these exergames, some have been specifically designed for stroke rehabilitation, including for balance training as well as for training for both lower and upper limb deficits (eg, Caren system, Lokomat, Armeo, and Jintronix [4,6,9,10]). The nature of the exergame interface makes it possible to modify the difficulty level of the exercises via various parameters such as intensity (time and repetition), visual feedback, speed, strength, and range of motion [4]. Progressing the exercise to ensure it remains challenging may motivate the user to complete the exercises [4]. However, there is no way to ensure that the movements made in the virtual environment are performed correspond to what is being trained. When performed in clinic, a health professional can ensure that the exercises are adjusted, and the movements are executed appropriately. However, at home, monitoring by a health professional needs to be considered to prevent compensatory movements during the exercises, inadequate choice of difficulty level, inappropriate completion of the exercises, and lack of motivation to pursue exercise program. The interfaces of the exergames do not allow automatic adjustment adapted to the person’s abilities and do not sufficiently detect compensations [4,6,9,10]. Thus, the follow-up by a therapist would make it possible to optimize the exergames’ benefits for the user (such as by adapting the difficulty parameters and by choosing games that are relevant for the user) and to help transfer motor learning from the virtual environment to activities of daily living.

To increase monitoring, a telerehabilitation system could allow such a follow-up through videoconferencing sessions between the user and the therapist. Such systems are increasingly used to provide remote rehabilitation services [11]. Its efficiency, compared with the standard treatment (face-to-face interventions), has been demonstrated, resulting in similar clinical results among participants diagnosed with stroke [12], therefore increasing accessibility to stroke rehabilitation services. Combining telerehabilitation with virtual reality (VirTele) could allow live sessions in which the therapist can observe the user playing and follow the game screen, at the same time, to assess how the user manages to complete the movements requested through the exercises (detect compensations, correct pathological patterns, and directly modify the difficulty setting necessary for the smooth running of the exercise). Therefore, the VirTele technology could allow continuous adjustment to the exercises and monitoring of the user completion to create a more personalized, tailored training program. Furthermore, in the long term, the VirTele exercise program could empower users to integrate the use of their impaired UE in their daily activities, through shared decisions made with the therapist.

### Study Objectives

Given the evidence for the efficacy of exergames as well as telerehabilitation for stroke rehabilitation, but the limited evidence of combining these technologies, such as in the VirTele program, the overall goals of this study are to explore its preliminary efficacy for UE rehabilitation and examine its feasibility for use with stroke survivors at home. More specifically, the objectives of this study are to (1) determine the preliminary efficacy of VirTele on UE motor recovery, function, quality of life, and motivation in participants with chronic stroke, compared with conventional therapy (GRASP); (2) examine the feasibility of using VirTele with participants diagnosed with chronic stroke at home; and (3) identify the obstacles and facilitators for the technology use by participants diagnosed with stroke and stroke therapists and understand the shared decision-making process.

It is hypothesized that the exergame-telerehabilitation program will lead to greater UE motor recovery than usual care (GRASP) in participants with chronic stroke. We also hypothesize that the exergame-telerehabilitation program will have a greater impact on function, quality of life, and motivation in participants with chronic stroke.

## Methods

### Study Design

This is a mixed method study design consisting of a randomized, blinded feasibility trial with an embedded multiple case study that will take place in Montreal, Canada. The randomized feasibility trial is a two-arm, single-blind trial design in which eligible participants will be randomly allocated to an experimental (VirTele for 8 weeks) or control, usual care group (GRASP for 8 weeks). The feasibility trial captures both feasibility and preliminary efficacy outcomes. Publishing the feasibility results would provide a better understanding of the context in which the efficacy data were collected and a better interpretation of the final results [13,14].

**Figure 1:**
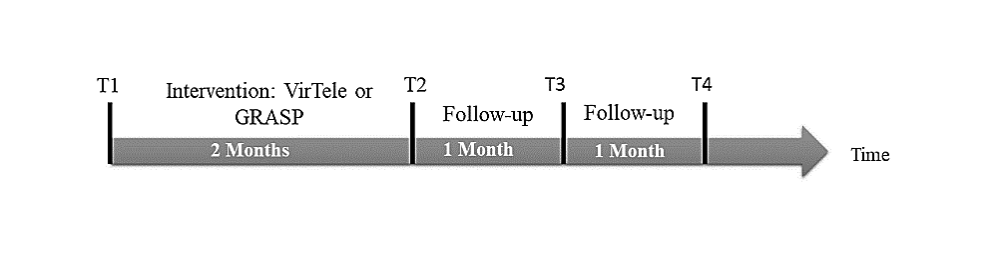
Description of the outcome measurement time. GRASP: Graded Repetitive Arm Supplementary Program; VirTele: program that combines virtual reality exergame and telerehabilitation application.

Outcome measures will be assessed for both groups on four occasions: at baseline (T1), at the end of 2-month intervention (T2), after a 1-month follow-up period (T3), and after a 2-month follow-up period (T4; Figure 1).

Randomization and evaluations will be performed by research assistants who are not involved in the study. All participants will provide informed written consent before enrollment. This study is registered at clinicaltrials.gov (NCT03759106) and has received ethics approval from the Research Ethics Boards of Centre for Interdisciplinary Research in Rehabilitation of Greater Montreal (June 28, 2018).

### Participant Selection and Recruitment Strategy

At study entry, we will administer the Chedoke-McMaster arm component [15] to get evidence of UE impairment. Only participants with a score of 2 to 6 will be eligible. We will also verify balance maintenance during sitting position and detect any UE mobility restrictions limiting the ability to play (restricted shoulder movements because of pain) through active and passive mobilizations.

Stroke survivors who have residual UE deficits and are no longer receiving rehabilitation services will be eligible for study participation if they fulfill the eligibility criteria. The inclusion criteria are as follows: first-time unilateral ischemic or hemorrhagic stroke or no residual deficits from a previous stroke and able to use the Jintronix system (eg, able to move the game avatar with impaired limb). The exclusion criteria are as follows: being medically unstable (eg, uncontrolled cardiac condition), severe cognitive or communication deficits, visual impairments limiting the ability to use the games, and UE mobility restrictions limiting the ability to play (eg, restricted shoulder movements because of pain).

We intend to recruit participants from the community and from the archives of rehabilitation centers (offline) situated in the Montreal, Sherbrooke, and Laval areas (Quebec, Canada).

Study therapists (physical therapists or occupational therapists) from the different participating sites will also be included in the study to explore their experiences with the technology and comprehend the shared decision-making process underlying their choice of games and difficulty levels.

### Sampling

To date, no studies have reported on the use of combining such technologies, such as in the VirTele program so that this randomized, blinded feasibility trial will provide evidence for the effect size. However, as the first estimate of effect size, a sample size of 52 participants has been calculated using the Fugl-Meyer Upper Extremity Assessment as a primary outcome and G*Power 3.1 [16]. We assumed a medium effect size of 0.2, which was reported in a study with chronic stroke survivors with a 2-arm randomized clinical trial [17] and accounting for 20% retention issues (alpha=.05 and power=80%). This sample size corresponds to recommendations for pilot efficacy and feasibility trials [18] and is realistic, given time and budgetary constraints as well as recruitment potential. Thus, for the randomized, blinded feasibility trial, 52 participants will be recruited and randomly allocated to the experimental (n=26) or control group (n=26). A block randomization with a block size of six will be used, given time and material constraints.

For the multiple case study component, four therapists and the first 10 participants from the experimental group will be invited to participate in interviews. However, the final sample size of the participants diagnosed with stroke will be adjusted depending on the qualitative data saturation [19].

### Description of Interventions

#### Experimental Group

The participants in the experimental group will receive the VirTele program. VirTele is an 8-week home program during which participants will use the Jintronix exergame [10] for UE training and the Reacts telerehabilitation application [20] to conduct videoconferenced sessions with the therapists (physical therapists or occupational therapists). All the equipment necessary for the proper functioning of the VirTele program, including the computer, the Kinect camera, the Reacts, and Jintronix software as well as a constant internet accessibility (USB internet key), will be provided for free to the participants. A technician will oversee the transport and the installation.

#### Telerehabilitation Component

The Reacts application is an interactive audio-video platform that allows secure communication between therapists and participants using standard computer or tablet technologies [20]. The application enables a live game access when it is used in combination with Jintronix. Thus, the therapist will be able to see the participant doing exercises and see the exergame platform while it is being used. The access to the live game platform allows the therapist to track the tasks or movements required by the game and see how the participant manages to accomplish them to adjust the difficulty level of the game according to the participant’s motor skills and interest. The Web sessions with Reacts will take place three times a week for 2 weeks, twice a week for 2 weeks, and then once a week for the remaining 4 weeks.

Reacts can also be used alone, making it possible for the participant to interact with the therapist when finishing the exercises. These sessions provide an opportunity to discuss difficulties regarding playing games and to subsequently modify the rehabilitation program according to the participant’s abilities and preferences. This shared decision-making process can help foster motivation to make health-related decisions (eg, setting goals weekly with the therapist, determine the optimal level of difficulty of the exercises, and choosing the games) and specially to continue rehabilitation after the end of the intervention. This shared decision-making process will aim to increase the empowerment of the participant, facilitating subsequently the transfer of the functional gains acquired in the context of the study into real life. This transfer occurs by identifying ways of reproducing the tasks of the game in activities of the daily life and increasing the use of the affected UE in the long term. Participant empowerment will be further encouraged by using motivational interviewing based on self-determination theory (SDT) principles during telerehabilitation sessions [21,22]. This theory states that humans naturally tend to achieve changes that respect and enable the satisfaction of their three basic psychological needs, namely, (1) autonomy, (2) connectivity, and (3) competence [22,23]. There are two regulation processes predicting behavioral engagement and maintenance: the behavior emanating from intrinsic motivation and the internalization of extrinsic motivation [23].

#### Virtual Reality Component

The Jintronix exergame consists of six UE games played for varying amounts of time and at different difficulty levels (speed, precision, and range of movements), which can be tracked remotely by the therapist asynchronously through the data provided in Jintronix Web-based portal. The therapist is also able to modify the difficulty parameters according to the performance data recorded on the portal. This exergame uses a Kinect camera, which captures the person’s body movements without wearing sensors. Participants will be invited to use Jintronix at least five times a week for 8 weeks, for 30-min sessions, performing a total of 20 hours of exercise. A minimum of 15 hours is suggested for an intervention to result in a moderate improvement in activities related to daily living following a stroke [6].

Jintronix includes an automated log system, which records the active time spent by participants in each game and the score achieved. The therapist can access the exergame interface at any time to monitor the participant’s progress and adherence to the exercise program and modify the difficulty level.

Before starting the intervention, therapists will receive training in motivational interviewing [24] to ensure a client-centered rehabilitation program that aligns with the SDT [21]. We used SDT [23] as a conceptual framework to guide the VirTele intervention to empower the participant and solicit their interest and motivation for the treatment plan that the therapist and participant will decide on together. The combination of the telerehabilitation system (Reacts) and the Jintronix exergaming system aims to foster participant-therapist interaction throughout the rehabilitation program and to develop a partnership relationship based on information sharing and trust. It is in this perspective that SDT was integrated. Its constructs were used as a basis for a discussion plan which the therapist refers to during videoconferenced sessions.

All participants in the experimental group will participate in a 30-min training session with the technician responsible for the installation of the technology to learn to use the VirTele technology.

#### Control Group

The participants in the control group will receive GRASP based on their UE function. It includes strengthening exercises of arm and hand, range of motion, and functional arm activities [5]. The program includes some equipment such as a ball, a bean bag, or paper clips. The participants will be invited to carry out the GRASP exercises over the 8 weeks, 5 days a week for 30 min each day, performing 20 hours of exercises in total (same as experimental group). A paper log journal will be provided to track the amount of time spent on the exercises and the number of sessions as well as any adverse events (fatigue and pain). The control group will not receive any follow-up with the therapist, but at the end of the study, the participants will be offered one session with the therapist to discuss strategies for improving long-term UE function. Before starting the intervention with GRASP, the participants will receive a 30-min training session for using the program equipment by one of the therapists included in the study.

### Data Collection

#### Quantitative Data

At study entry, the participant’s sociodemographic information (gender, age, civil status, language, number of years of education completed, primary occupation, and stroke characteristics) will be collected for descriptive purposes.

For the randomized, blinded feasibility trial, several outcomes measures will be used to address the different objectives. The first objective of the trial is to determine the preliminary efficacy of VirTele on UE motor recovery, function, quality of life, and motivation, in participants with chronic stroke, compared with conventional therapy (GRASP).

The Fugl-Meyer Upper Extremity Assessment will be the primary outcome to determine the efficacy of the technology for UE motor control recovery. This is a performance-based measure of UE impairment [25,26]. It includes 13 items scored on a 3-point ordinal scale of 0 to 2 [26]. The Fugl-Meyer Upper Extremity Assessment has been shown to have good internal consistency (alpha=.82-.84) and good concurrent validity (r=0.74) [25].

The secondary outcomes are self-reported questionnaires and include the Motor Activity log 28 [27,28], the Stroke Impact Scale-16 [29,30], and the Treatment Self-Regulation Questionnaire-13 [31].

The Motor Activity log 28 is a self-reported measure of UE use [27,28]. This rates the quality and frequency of use of the UE in 28 everyday tasks and is administered by interview [27,28]. The Motor Activity log 28 demonstrated high reliability (r=0.82) and high validity, with excellent concurrent correlation with Stroke Impact Scale hand function scores (r=0.72) [32]. The impact on quality of life will be determined using the Stroke Impact Scale-16, a stroke-specific, self-reported, health status measure consisting of 16 items concerning daily activities [29,30]. The Stroke Impact Scale-16 has been shown to have good internal consistency (alpha=.87) and a good convergent and discriminant validity [33]. Motivation will be measured using the Treatment Self-Regulation Questionnaire-13 [31], a 13-item questionnaire that has been developed to measure treatment motivation, aligned with SDT. The Treatment Self-Regulation Questionnaire has been shown to be reliable with a high internal consistency (alpha=.73-.95) and valid across health care contexts and has been used in rehabilitation [34]. The two regulation processes of the SDT (intrinsic and extrinsic motivation) are targeted in the form of subscales in the Treatment Self-Regulation Questionnaire-13 [31]. The use of this questionnaire allows us to investigate the impact that the motivation could have on adherence to the program and its effectiveness.

The second objective of the randomized, blinded feasibility trial is to assess the feasibility of using VirTele with participants diagnosed with stroke at home. Feasibility data include indicators of process, resources, management, and scientific feasibility [35]. In Table 1, we describe all the outcomes that will be used for each indicator. These data will also provide evidence to examine the validity of the research protocol to inform the planning of a larger clinical trial.

A trained assessor who is not involved in the delivery of interventions and blinded to group assignment will be responsible for the face-to-face administration of the outcome measures.

**Table 1:** Description of feasibility indicators outcomes.

Feasibility indicators		Outcomes
**Process**		
	Recruitment rate	Percentage of participants who meet the eligibility criteria and accept to participate (20%) Rate of participants per month Duration of recruitment
	Retention rate	Percentage of participants who complete the 2-month intervention with telerehabilitation with virtual reality
**Resources**		
	Exercise adherence rate	Percentage of participants who complete 150 min of Jintronix exercises per week
	Number and duration of sessions	These data will be obtained from the Jintronix exergame and Reacts app system
	Frequency and time spent by the therapist assisting for real-time sessions with Jintronix	Logs completed by the therapist at the end of each session
	Resources utilization	Logs completed and time spent by therapists and technical staff
**Management**		
	Technical problems with the technology	Obtained from a log maintained by the study therapists and technical team
	The role of the shared decision making and empowerment in achieving task goals	Encourage participants to report new task goals every week Percentage of participants who achieved the goals set with the therapist Percentage of task goals achieved per participant
**Scientific**		
	Safety	Occurrence of adverse events (pain, falls, motion sickness, dizziness, exertion, fatigue, and headaches) will be documented by a computerized participant log
	Satisfaction	With the technology: the Modified Short Feedback Questionnaire [36] With the interaction between the therapist and the participant: Health Care Climate Questionnaire (Perceived Autonomy Support) [37]
	Size of sample	The calculation will be done from the size of the treatment effect or the variance of the treatment effect

#### Qualitative Data

Individual semistructured interviews of 30 min will be conducted with the first 10 participants from the experimental group (n=10) after the end of 8-week intervention. The interview guide will be developed based on the Unified Theory of Acceptance and Use of Technology (UTAUT) conceptual framework [38]. This theory includes four essential constructs (expected performance, expected effort, social influence, and facilitating conditions), which are considered as direct determinants of the intention and behavior of adoption and the use of new technologies by stakeholders [38]. Therefore, semistructured interviews will be used to inform not only the intention and behavior of adoption and use of VirTele by participants diagnosed with stroke and therapists but also of UE use in the future (through UTAUT constructs). When combined with SDT constructs, the interview will inform the participants’ empowerment and the shared decision-making process underlying the therapist choice of games and difficulty levels.

The study therapists will also be interviewed to describe their experience with VirTele (obstacles and facilitators), explore their behavior to see if they align with SDT, and investigate the shared decision-making process underlying their choice of games and difficulty levels. Individual semistructured interviews of 30 min will be used. The UTAUT and the SDT will serve as basis for the interview guide development.

All interviews will be voice recorded and transcribed verbatim. A logbook and reflective notes will also be taken.

### Data Analysis

#### Quantitative Data Analyses

Statistical analysis of the quantitative data will be performed using the Statistica software. Descriptive statistics will be used to report sociodemographic characteristics of participants (age, gender, handedness, and stroke characteristics) in both groups (experimental vs control). To address the first objective of the randomized, blinded feasibility trial, a mixed model approach will be applied for primary and secondary outcomes measure. Each model will contain one between-subject effect factor (group: control vs experimental), one within-subject effect factor (time: T1, T2, T3, and T4), and two covariates (gender and age) factors, which may impact exergame use. For each outcome measures, residual plots will be examined to verify normality and identify the best covariance structure. All the outcomes measure changes will be compared with the minimal clinically important differences. The effect size of the comparison between experimental and control groups will be calculated to estimate the final sample size. To address the second objective of the randomized, blinded feasibility trial, descriptive statistics (frequencies, means, and standard deviations) will be used to highlight the amount of exercise performed, the occurrence of adverse events, and participant and therapist satisfaction level in the experimental and control groups.

#### Qualitative Data Analyses

For the qualitative data, thematic analysis will be conducted for each case group. Transcripts will be coded using a predetermined coding scheme (Multimedia Appendix 1) based on the conceptual frameworks as well as other codes emerging from the data using NVivo software, and then codes will be grouped into overarching themes. To ensure the scientific rigor of qualitative data, the principles of Lincoln and Guba [39] will be applied. Audit trail and verification by members will be done to respect confirmability. A debriefing (external verification) will be applied to ensure credibility. Reliability will be achieved with verification by two coders of a part of the data. Transferability will be assured by taking reflexive notes and a detailed description of the context of the intervention. During the analysis, the results of the qualitative and quantitative data will be compared to help explain findings.

## Results

We expect to (1) collect preliminary efficacy data of this technology on the functional and motor recovery of the UE, following a stroke; (2) collect feasibility data with users at home (adherence, safety, and technical difficulties); and (3) identify the obstacles and facilitators for the technology use and understand the shared decision-making process during the VirTele program.

At the time of this manuscript submission, data collection was ongoing with one participant who had completed the study (experimental group) and used VirTele for 2 months [40] (Multimedia Appendix 2). At this stage of the study, we have not yet started the data analysis.

## Discussion

### Study Design

This paper describes the research protocol for a mixed method study, including a randomized, blinded feasibility trial with an embedded multiple case study. The aims of this study are to (1) determine the preliminary efficacy of VirTele on UE motor recovery, function, quality of life, and motivation in participants with chronic stroke, compared with conventional therapy (GRASP); (2) examine the feasibility of using VirTele with participants diagnosed with chronic stroke at home; and (3) identify the obstacles and facilitators for the technology use by participants diagnosed with stroke and stroke therapists and understand the shared decision-making process.

We have chosen a mixed study design because the use of both qualitative and quantitative approaches makes it possible to construct a more complete image of the studied phenomenon. The combination of the two methodologies should be approached not from the point of view of their differences but from the complementarities they can bring to the study [41]. If the feasibility trial examines the content of the intervention to see if it is effective and feasible, the qualitative approach examines the context of the intervention to see if it can be accepted and applied in clinical practice and explain some of the quantitative findings [42]. Feasibility trials are important to ensure that larger randomized clinical trials are rigorous and feasible and economically justifiable [13].

### Data Collection

Participants will be recruited from different sites to increase the representativeness of the target population in the region. The control group will not receive any motivational interviewing or follow-up to keep the standard aspect of therapy that corresponds most to the clinical reality. This will enable us to identify the added value of the VirTele program compared with GRASP. After outcome measures collection in T1 and T2, to compare the effect of each intervention within and between groups, we will collect additional measures at T3 and T4 to evaluate the retention of gains.

The multiple case study provides an in-depth description of stakeholders use experience and potential use of VirTele program. In this study, we will have two case groups: participants diagnosed with stroke and study therapists (physical therapists or occupational therapist). Each of these cases experiences the intervention differently, and it is therefore essential to report them through interviews. The variation of the cases makes it possible to increase the variation of the experiences and thus to increase the robustness of the qualitative results [19]. UTAUT and SDT will serve as a basis for establishing certain links between the concepts that will emerge (Multimedia Appendix 1).

The results of this study will allow to verify if all elements of the protocol work well together to conduct a broader future study. We have tried to provide as much detail as possible about the various processes and steps of the protocol to facilitate its reproduction by other studies that seek to develop tools for the remote management of chronic diseases.

This project will also provide preliminary evidence of the efficacy of VirTele on motor and functional recovery of the UE following chronic stroke for future guidelines review, although studies caution us about solely using results from feasibility studies to establish intervention efficacy [35].

### Conclusions

Extending rehabilitation following a stroke with remote services may be a promising strategy to overcome the limited resources in the health system. The VirTele program is a new approach that may provide stroke survivors continuous and remote access to rehabilitation services. This paper describes the protocol underlying the study of this technology to better understand how it can be used among different stakeholders and explore its preliminary efficacy in a chronic stroke population.

## Appendix

Multimedia Appendix 1 The predetermined qualitative coding scheme.

Multimedia Appendix 2 A screenshot of the combined use of Jintronix and Reacts.

Multimedia Appendix 3 Peer-review reports from the Canadian Institutes of Health Research - Part 1.

Multimedia Appendix 4 Peer-review reports from the Canadian Institutes of Health Research - Part 2.

## Abbreviations

GRASP: Graded Repetitive Arm Supplementary Program
SDT: self-determination theory
T1: at baseline
T2: at the end of 2-month intervention
T3: after a 1-month follow-up period
T4: after a 2-month follow-up period
UE: upper extremity
UTAUT: Unified Theory of Acceptance and Use of Technology
VirTele: program that combines virtual reality exergame and telerehabilitation application

## References

[1] Nichols-Larsen DS, Clark PC, Zeringue A, Greenspan A, Blanton S (2005). Factors influencing stroke survivors' quality of life during subacute recovery. Stroke.

[2] Casaubon LK, Boulanger J, Glasser E, Blacquiere D, Boucher S, Brown K, Goddard T, Gordon J, Horton M, Lalonde J, LaRivière C, Lavoie P, Leslie P, McNeill J, Menon BK, Moses B, Penn M, Perry J, Snieder E, Tymianski D, Foley N, Smith EE, Gubitz G, Hill MD, Lindsay P, Heart and Stroke Foundation of Canada Canadian Stroke Best Practices Advisory Committee (2016). Canadian stroke best practice recommendations: acute inpatient stroke care guidelines, update 2015. Int J Stroke.

[3] Lindsay M, Bayley M, Hellings C, Hill M, Woodbury E, Phillips S (2008). Canadian best practice recommendations for stroke care (updated 2008). Can Med Assoc J.

[4] Weiss PL, Keshner EA, Levin MF (2014). Virtual Reality for Physical and Motor Rehabilitation.

[5] Harris JE, Eng JJ, Miller WC, Dawson AS (2009). A self-administered Graded Repetitive Arm Supplementary Program (GRASP) improves arm function during inpatient stroke rehabilitation: a multi-site randomized controlled trial. Stroke.

[6] Laver KE, Lange B, George S, Deutsch JE, Saposnik G, Crotty M (2017). Virtual reality for stroke rehabilitation. Cochrane Database Syst Rev.

[7] McCabe J, Monkiewicz M, Holcomb J, Pundik S, Daly JJ (2015). Comparison of robotics, functional electrical stimulation, and motor learning methods for treatment of persistent upper extremity dysfunction after stroke: a randomized controlled trial. Arch Phys Med Rehabil.

[8] Ward NS, Brander F, Kelly K (2019). Intensive upper limb neurorehabilitation in chronic stroke: outcomes from the Queen Square programme. J Neurol Neurosurg Psychiatry.

[9] Subramanian SK, Lourenço CB, Chilingaryan G, Sveistrup H, Levin MF (2013). Arm motor recovery using a virtual reality intervention in chronic stroke: randomized control trial. Neurorehabil Neural Repair.

[10] Jintronix.

[11] Blacquiere D, Lindsay MP, Foley N, Taralson C, Alcock S, Balg C, Bhogal S, Cole J, Eustace M, Gallagher P, Ghanem A, Hoechsmann A, Hunter G, Khan K, Marrero A, Moses B, Rayner K, Samis A, Smitko E, Vibe M, Gubitz G, Dowlatshahi D, Phillips S, Silver FL, Heart and Stroke Foundation Canadian Stroke Best Practice Committees (2017). Canadian stroke best practice recommendations: telestroke best practice guidelines update 2017. Int J Stroke.

[12] Cikajlo I, Rudolf M, Goljar N, Burger H, Matjačić Z (2012). Telerehabilitation using virtual reality task can improve balance in patients with stroke. Disabil Rehabil.

[13] Whitehead AL, Sully BG, Campbell MJ (2014). Pilot and feasibility studies: is there a difference from each other and from a randomised controlled trial?. Contemp Clin Trials.

[14] Bowen DJ, Kreuter M, Spring B, Cofta-Woerpel L, Linnan L, Weiner D, Bakken S, Kaplan CP, Squiers L, Fabrizio C, Fernandez M (2009). How we design feasibility studies. Am J Prev Med.

[15] Parry R (1996). Chedoke-McMaster stroke assessment — development, validation and administration manual. Physiother.

[16] G*Power: Statistical Power Analyses for Windows and Mac.

[17] Pang MY, Harris JE, Eng JJ (2006). A community-based upper-extremity group exercise program improves motor function and performance of functional activities in chronic stroke: a randomized controlled trial. Arch Phys Med Rehabil.

[18] Julious SA (2005). Sample size of 12 per group rule of thumb for a pilot study. Pharm Stat.

[19] Fortin MF, Gagnon J (2010). Foundations and Stages of the Research Process: Quantitative and Qualitative Methods. Second Edition. Book in French. Fondements et étapes du processus de recherche: méthodes quantitatives et qualitatives 2e éd.

[20] IIT Reacts.

[21] Vansteenkiste M, Niemiec C, Soenens B, Urdan T, Karabenick S (2010). The development of the five mini-theories of self-determination theory: an historical overview, emerging trends, and future directions. The Decade Ahead: Theoretical Perspectives on Motivation and Achievement.

[22] Sweet SN, Rocchi M, Arbour-Nicitopoulos K, Kairy D, Fillion B (2017). A telerehabilitation approach to enhance quality of life through exercise among adults with paraplegia: study protocol. JMIR Res Protoc.

[23] Deci EL, Ryan RM (2000). The 'What' and 'Why' of goal pursuits: human needs and the self-determination of behavior. Psychol Inq.

[24] Miller WR, Rollnick S (2012). Motivational Interviewing: Helping People Change. Third Edition.

[25] Page SJ, Hade E, Persch A (2015). Psychometrics of the wrist stability and hand mobility subscales of the Fugl-Meyer assessment in moderately impaired stroke. Phys Ther.

[26] Singer B, Garcia-Vega J (2017). The Fugl-Meyer upper extremity scale. J Physiother.

[27] Lang CE, Edwards DF, Birkenmeier RL, Dromerick AW (2008). Estimating minimal clinically important differences of upper-extremity measures early after stroke. Arch Phys Med Rehabil.

[28] van der Lee JH, Beckerman H, Knol DL, de Vet HC, Bouter LM (2004). Clinimetric properties of the motor activity log for the assessment of arm use in hemiparetic patients. Stroke.

[29] Chou C, Ou Y, Chiang T (2015). Psychometric comparisons of four disease-specific health-related quality of life measures for stroke survivors. Clin Rehabil.

[30] Fulk GD, Ludwig M, Dunning K, Golden S, Boyne P, West T (2010). How much change in the stroke impact scale-16 is important to people who have experienced a stroke?. Top Stroke Rehabil.

[31] Levesque CS, Williams GC, Elliot D, Pickering MA, Bodenhamer B, Finley PJ (2007). Validating the theoretical structure of the Treatment Self-Regulation Questionnaire (TSRQ) across three different health behaviors. Health Educ Res.

[32] Uswatte G, Taub E, Morris D, Light K, Thompson PA (2006). The Motor Activity Log-28: assessing daily use of the hemiparetic arm after stroke. Neurology.

[33] Edwards B, O'Connell B (2003). Internal consistency and validity of the Stroke Impact Scale 2.0 (SIS 2.0) and SIS-16 in an Australian sample. Qual Life Res.

[34] Chan DK, Lonsdale C, Ho PY, Yung PS, Chan KM (2009). Patient motivation and adherence to postsurgery rehabilitation exercise recommendations: the influence of physiotherapists' autonomy-supportive behaviors. Arch Phys Med Rehabil.

[35] Thabane L, Ma J, Chu R, Cheng J, Ismaila A, Rios LP, Robson R, Thabane M, Giangregorio L, Goldsmith CH (2010). A tutorial on pilot studies: the what, why and how. BMC Med Res Methodol.

[36] Davis FD (1989). Perceived Usefulness, Perceived ease of use, and user acceptance of information technology. Manag Inf Syst Q.

[37] Gremigni P, Sommaruga M, Peltenburg M (2008). Validation of the Health Care Communication Questionnaire (HCCQ) to measure outpatients' experience of communication with hospital staff. Patient Educ Couns.

[38] Williams MD, Rana NP, Dwivedi YK (2015). The unified theory of acceptance and use of technology (UTAUT): a literature review. J Enterp Inf Manag.

[39] Guba EG, Lincoln YS (1985). Naturalistic Inquiry.

[40] Allegue DR, Kairy D, Higgins J, Archambault P, Michaud F, Miller W, Sweet S, Tousignant M (2019). Remote Rehabilitation Training Using the Combination of an Exergame and Telerehabilitation Application: A Case Report of an Elderly Chronic Stroke Survivor. Proceedings of the 2019 International Conference on Virtual Rehabilitation.

[41] Levy R (1997). Reflection on Public Health Research: From Metaphors to the Rescue. Article in French. Réflexion sur la recherche en santé publique:des métaphores à la rescousse. Ruptures.

[42] Albright K, Gechter K, Kempe A (2013). Importance of mixed methods in pragmatic trials and dissemination and implementation research. Acad Pediatr.

