# Supplementary material for: Optimization of Upper Extremity Rehabilitation by Combining Telerehabilitation With an Exergame in People With Chronic Stroke: Protocol for a Mixed Methods Study
Source: JMIR Res Protoc. 2020 May 21;9(5):e14629. doi: 10.2196/14629 (PMC7273231; doi:10.2196/14629)
Supplement: Multimedia Appendix 1 [file resprot_v9i5e14629_app1.pdf]

Themes	Main codes	Explanation of main codes	
		Participant	Therapist
**Experience with VirTele exergame system**			
	Performance	Of the affected upper extremity<br>• perceived change in arm functional performance / impact on activities of daily life compared to expectations<br><br>Of the technology<br>• expected benefits<br>• compatibility with needs / expectations<br>• obstacles or technical problems	• quality of interactions with technology<br>• services rendered<br>• compatibility with needs / expectations<br>• obstacles or technical problems
	Effort	• apprehensions<br>• expected ease of use/ actual effort required to use the technology<br>• compatibility with needs / expectations	
	Facilitating conditions	• attitude towards the use of technology<br>• resources put in place<br>• ease with technology	
**Empowerment of the participant**			
	Participation in shared decision-making	• role during the consultation<br>• experience with the guidance provided<br>• goals fixed / patterns of achievement	• responsibilities and role during the intervention

Management of care	• management of the consultations and games sessions<br>• resources put in place/ support<br>• comparison with previous rehabilitation experience	• the logic behind the choice of games, the adjustment of the difficulty level, number of repetitions and any other parameters
Participant states and capacities	• perceived personal control over health and healthcare<br>• perceived general health condition after the end of the program<br>• perceived motivation and autonomy support	• transfer into activities of daily living<br>• strategies used to support participant autonomy
