# Supplementary figures and images for: Optimization of Upper Extremity Rehabilitation by Combining Telerehabilitation With an Exergame in People With Chronic Stroke: Protocol for a Mixed Methods Study
Source: JMIR Res Protoc. 2020 May 21;9(5):e14629. doi: 10.2196/14629 (PMC7273231; doi:10.2196/14629)

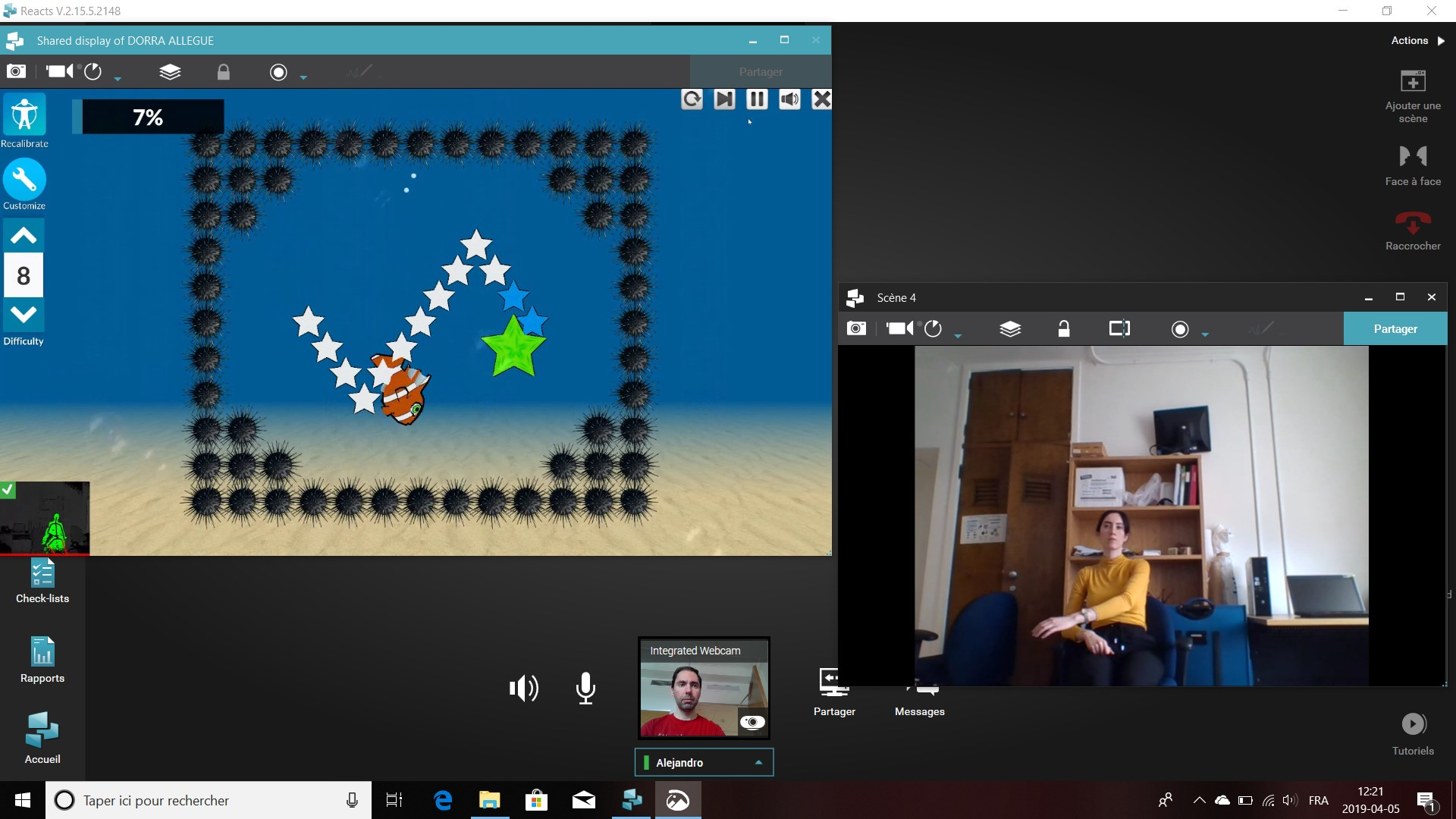

Supplement: Multimedia Appendix 2 [file resprot_v9i5e14629_app2.png]
