# Supplementary material for: Optimization of Upper Extremity Rehabilitation by Combining Telerehabilitation With an Exergame in People With Chronic Stroke: Protocol for a Mixed Methods Study
Source: JMIR Res Protoc. 2020 May 21;9(5):e14629. doi: 10.2196/14629 (PMC7273231; doi:10.2196/14629)
Supplement: Multimedia Appendix 3 [file resprot_v9i5e14629_app3.pdf]

Review Type/Type d'évaluation: Committee Member 1/Membre de comité 1
Name of Applicant/Nom du chercheur: Kairy, Dahlia
Application No./Numéro de demande: 385297
Agency/Agence: CIHR/IRSC
Competition/Concours: 2017-06-13 Catalyst Grant: Personalized Health Catalyst Grants/Subvention catalyseur: Subventions Catalyseur sur la santé personnalisée
Committee/Comité: Personalized Health - novel e-health applications/santé personnalisée - applications de cybersanté novatrices
Title/Titre: Optimizing a home-based virtual reality exercise program for chronic stroke patients: A telerehabilitation approach

---

Assessment/Évaluation:

Dahlia Kairy (Université de Montréal)

Optimizing a home-based virtual reality exercise program for chronic stroke patients: A telerehabilitation approach

A brief synopsis of the proposal

The authors have used the Jintronix video game system for upper extremity rehabilitation in chronic stroke patients living at home. Using this VR system, the clinician obtained information about the patient's performance on the games, and modified exercise programs remotely. They have also examined the clinical implementation in Quebec rehabilitation centers of the Reacts App, an interactive audio-video platform. For this study, they are combining these two: the Jintronix video game system and the Reacts App - the VirTele (Virtual Reality Telerehabilitation). The Vir-Tele program for chronic stroke rehabilitation can allow for engaging upper limb rehabilitation programs which are adequately monitored and tailored to the person's progress and preferences. This project aims to provide evidence regarding the program's clinical efficacy in terms of function, social participation and quality of life, and determine the feasibility, and acceptability (from the patient and therapist's perspective).

They will performs a feasibility randomized clinical trial with 52 participants who have suffered a stroke, have residual upper extremity, have completed their rehabilitation and are living in the community. Participants will be randomized to (1) an 8-week home-based VR exercise program with periodic real-time online monitoring by a clinician or (2) usual care. Four evaluation time-points are planned: at baseline, after the two-month intervention or control period and one and two months later. Qualitative interviews with study participants as well as clinicians will also be conducted

-

Assessment of the proposal

This approach is very novel as it uses virtual reality as well as an App to deliver the intervention. The team

Review Type/Type d'évaluation: Committee Member 1/Membre de comité 1
Name of Applicant/Nom du chercheur: Kairy, Dahlia
Application No./Numéro de demande: 385297
Agency/Agence: CIHR/IRSC
Competition/Concours: 2017-06-13 Catalyst Grant: Personalized Health Catalyst Grants/Subvention catalyseur: Subventions Catalyseur sur la santé personnalisée
Committee/Comité: Personalized Health - novel e-health applications/santé personnalisée - applications de cybersanté novatrices
Title/Titre: Optimizing a home-based virtual reality exercise program for chronic stroke patients: A telerehabilitation approach

---

Assessment/Évaluation:

has performed work on this before and completed an RCT with the virtual reality component. This study builds on their work.

The RCT is well designed and there is justified sample size calculation. The measures are well thought out. The intervention is based on theory as well as the results of their previous trial. The only weakness is that one of the purposes of this study is to determine the sample size for a future RCT, yet this study is powered to detect a difference. Should this study have only negative results, is a larger RCT warranted?

The KT-plan is well thought out

Team: no concerns

<u>Sex/Gender Consideration</u>

The applicants' state that "while there are no know sex or gender related factors which have been shown to impact on recovery post stroke, video game use differed between men and women. Reasons for choosing to use video games and types of games played differ. Therefore, during the data collection, in particular the qualitative aspects of the study, specific questions will be aimed at trying to understand what motivates the participant to choose or not to choose to do the exercise program on a regular basis. In the data analysis plan, gender is considered as a covariant.
