# Supplementary material for: Optimization of Upper Extremity Rehabilitation by Combining Telerehabilitation With an Exergame in People With Chronic Stroke: Protocol for a Mixed Methods Study
Source: JMIR Res Protoc. 2020 May 21;9(5):e14629. doi: 10.2196/14629 (PMC7273231; doi:10.2196/14629)
Supplement: Multimedia Appendix 4 [file resprot_v9i5e14629_app4.pdf]

Review Type/Type d'évaluation: Committee Member 2/Membre de comité 2
Name of Applicant/Nom du chercheur: Kairy, Dahlia
Application No./Numéro de demande: 385297
Agency/Agence: CIHR/IRSC
Competition/Concours: 2017-06-13 Catalyst Grant: Personalized Health Catalyst Grants/Subvention catalyseur: Subventions Catalyseur sur la santé personnalisée
Committee/Comité: Personalized Health - novel e-health applications/santé personnalisée - applications de cybersanté novatrices
Title/Titre: Optimizing a home-based virtual reality exercise program for chronic stroke patients: A telerehabilitation approach

---

Assessment/Évaluation:

Kairy - 385297

The purpose of the study is to build in periodic patient-therapist real-time interaction to boost patient motivation, ensure the exercises are progressed appropriately for the patient's abilities and ensure long-term increased use of the upper-limb.

The project aims to provide evidence regarding the program's clinical efficacy in terms of function, social participation and quality of life, and determine the feasibility (e.g. compliance, technical difficulties, safety), and acceptability (from the patient and therapist's perspective).

The applicants have used the Jintronix video game system for upper extremity rehabilitation in chronic stroke patients living at home. And the use of the Reacts app to ensure virtual reality exercise programs are monitored and tailor to a patients experience. Group have received local funding to work with the App.

- In terms of study design, participants will be randomized to (1) an 8-week home-based VR exercise program with periodic real-time online monitoring by a clinician or (2) usual care. Four evaluation time-points are planned: at baseline (T0), after the two-month intervention or control period (T1) and one and two months later (T2, T3).

- Qualitative interviews with study participants as well as clinicians will also be conducted, to provide more insight into how this novel intervention could be integrated into clinical practice, the extent to which this intervention addresses the users' needs, and its perceived impact on the shared decision-making process, participant empowerment and motivation to adopt new behaviors.

- Unclear how Self-Determination Theory is being operationalized in the proposal.

- Furthermore, how will Unified Theory of Acceptance and the Use of Technology Model be used to inform patient empowerment? Assumes it can explain behaviours as a mediator?

- Sample size calculation does not list the primary outcome. Safe to assume this is a subjective functional measure? Is there a clinically relevant value to shoot for? A minimal clinically important difference (MCID)?

**Review Type/Type d'évaluation:**	Committee Member 2/Membre de comité 2
**Name of Applicant/Nom du chercheur:**	Kairy, Dahlia
**Application No./Numéro de demande:**	385297
**Agency/Agence:**	CIHR/IRSC
**Competition/Concours:**	2017-06-13 Catalyst Grant: Personalized Health Catalyst Grants/Subvention catalyseur: Subventions Catalyseur sur la santé personnalisée
**Committee/Comité:**	Personalized Health - novel e-health applications/santé personnalisée - applications de cybersanté novatrices
**Title/Titre:**	Optimizing a home-based virtual reality exercise program for chronic stroke patients: A telerehabilitation approach

Assessment/Évaluation:

- How will visual impairment be assessed over the phone with respect to inclusion/exclusion?

- In terms of alignment with the catalyst grant, objective 3 is most closely aligned. The grant appears to have been proposed from an efficacy standpoint with the qualitative follow-up approach as an add on. The collection and use of qualitative data therefore requires more thought and detail as it will be critical for informing empowerment and shared decision making for the patients.

- How will the quantitative data from the two questionnaires (Modified Short Feedback an Health Care Climate) be used with the findings from the qualitative component? A true mixed-methods approach would integrate these findings. Details are lacking.

- The integration of sex and gender appears to be within the statistical model (gender and age as covariates). Why is gender considered a covariate? Sex could certainly be viewed as a moderator but fail to see how gender fits in.

- Main application document would benefit from a clear outline of roles and responsibilities for the investigators. As written, the team is primarily responsible for recruitment.

There is certainly novelty in the mode of patient care and likely has potential to impact recovery post stroke. However, the feasibility of use for this technology within the current model of service delivery may be prohibitive. Economic analysis will be required.
